# Current progress in research focused on salt tolerance in *Vitis vinifera* L.

**DOI:** 10.3389/fpls.2024.1353436

**Published:** 2024-02-08

**Authors:** Yan Han, Xiujie Li

**Affiliations:** Shandong Academy of Grape, Ji’nan, Shandong, China

**Keywords:** gene mining, germplasm resources, grape, salt tolerance, variety

## Abstract

Soil salinization represents an increasingly serious threat to agronomic productivity throughout the world, as rising ion concentrations can interfere with the growth and development of plants, ultimately reducing crop yields and quality. A combination of factors is driving this progressive soil salinization, including natural causes, global climate change, and irrigation practices that are increasing the global saline-alkali land footprint. Salt stress damages plants both by imposing osmotic stress that reduces water availability while also inducing direct sodium- and chlorine-mediated toxicity that harms plant cells. *Vitis vinifera* L. exhibits relatively high levels of resistance to soil salinization. However, as with other crops, grapevine growth, development, fruit yields, and fruit quality can all be adversely affected by salt stress. Many salt-tolerant grape germplasm resources have been screened in recent years, leading to the identification of many genes associated to salt stress and the characterization of the mechanistic basis for grapevine salt tolerance. These results have also been leveraged to improve grape yields through the growth of more tolerant cultivars and other appropriate cultivation measures. The present review was formulated to provide an overview of recent achievements in the field of research focused on grapevine salt tolerance from the perspectives of germplasm resource identification, the mining of functional genes, the cultivation of salt-tolerant grape varieties, and the selection of appropriate cultivation measures. Together, we hope that this systematic review will offer insight into promising approaches to enhancing grape salt tolerance in the future.

## Introduction

23% of the cultivated arable lands are saline over 100 countries in all continents. 20% (45 million ha) irrigated lands are human-induced salt-affected soils (secondary salinization) in the world (Zaman et al., 2018). For instance, 2.6×10^7^ hectares (ha) of the total land area are salt affected mainly in the north part and tidal coastal regions and 6.7 million ha lands of the irrigated areas are affected by secondary salinization in China. It is thought that productivity enhancement of salt-affected lands in irrigated areas is one important method to provide more food, fruit, feed, and fiber to the expanding population worldwide (Qadir et al., 2014).

**Figure 1:**
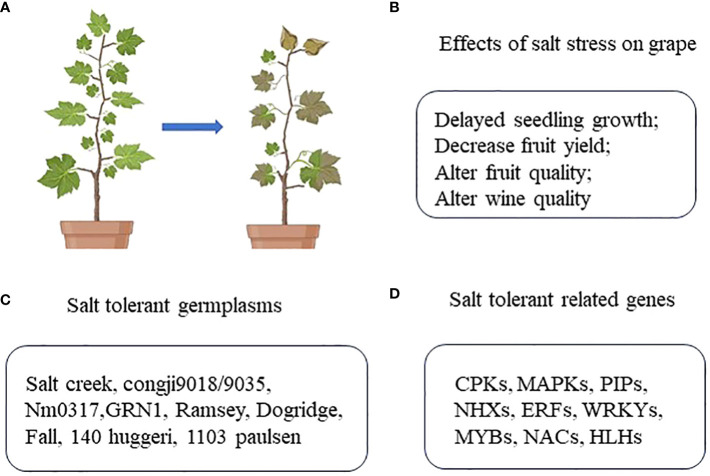
BBB model schema and cell culture and characterization. The proposed BBB model comprised of rat brain astrocytes (RBAs), rat brain microvascular endothelial cells (RBMECs) and rat brain pericyte cells (RBPs). RBAs were characterized by GFAP ICC with an expression of 93.25∓3.58, RBMECs were characterized by VEGFR2 ICC with an expression of 90.96∓6.51 and pericyte cells were characterized by PDGFR b ICC with an expression of 90.82∓3.85.

*Vitis* L. is among the most highly valued genera in the Vitaceae family, as the fruits it produces are rich in polyphenols and resveratrol that reportedly exhibit anti-aging activity. In addition to being consumed as fresh fruits, these grapes are also processed to produce raisins, juice, sauces, vinegar, and wine, all of which are important to the global fruit trade. Grapevines are plant species with a relatively high level of salt tolerance. Despite this advantageous trait, progressive soil salinization can still adversely impact the growth, fruit yield, and fruit quality (i.e. flavor, sugar content etc.), and further negatively affecting wine quality. The most effective approaches to improving grape production in lightly salinized soil and enhancing the growth and fruit quality of grapevines cultivated in the presence of soil salinization have thus been a focus of intensive research interest in recent years. This review was compiled in an effort to gain insight into the most effective means of improving the salt tolerance of grape plants, offering a theoretical reference for efforts to better explore the mechanistic basis for such salt tolerance, cultivate salt-tolerant grape varieties, and improve overall grape growth in saline soil (**Figure 1**).

## Studies of salt-tolerant grape germplasm resources

Germplasm resources serve as the basic materials for the breeding of novel cultivars while also enabling biotechnological research and germplasm innovation. Certain varieties of wild grapes exhibit high levels of adaptability and resistance to a variety of stressors, and thus represent valuable resources that can be used to broaden the gene pool as a means of expanding the genetic repertoire of cultivated grape varieties (Aradhya et al., 2003; Wan et al., 2008). Prior studies have established grapes as being moderately sensitive to salt stress (Maas and Hoffman, 1977). Salt-treated leaves generally exhibit higher chloride levels than sodium levels during various stages of growth, with this difference being elevated by an order of magnitude in leaves exhibiting stress symptoms. The ability of grape plants to minimize chloride accumulation has thus been used as a criterion when attempting to screen for salt-tolerant germplasm resources (Fort et al., 2013). The chloride exclusion abilities of *V. acerifolia*, *V. arizonica*, *V. berlandieri*, *V. doaniana*, and *V. girdiana* accessions gathered from the southwestern United States have been categorized and compared to the benchmark chloride excluder *V. rupestris* ‘St. George’ (Heinitz et al., 2014). Longii 9018/9035, NM:03-17, and GRN1 with lower chloride accumulation in leaves were identified as chloride excluders from among 16 grape rootstocks treated with 75 mM NaCl for three weeks, highlighting a clear relationship between the degree of fine root production and chloride accumulation (Bent, 2017). Approximately 60 wild *Vitis* species have been identified in four main eco-geographic regions of China, but their relative levels of salt tolerance remain to be assessed (Wan et al., 2008). Currently, the Ramsey and Dogridge rootstocks of the *V. champinii* species, as well as the Fall grape, 140 Ruggeri, and 1103 Paulsen progenies of the *V. berlandieri* × *V. rupestris* cross have been established as salt-tolerant germplasms such that they are commonly used as rootstocks when seeking to breed novel salt-tolerant cultivars (Zhou-Tsang et al., 2021).

Given the long history of grape cultivation throughout the globe together with the diverse range of complex hybrids and rootstocks generated through vegetative propagation, anywhere from 6,000 to 10,000 grape cultivars are thought to exist, providing ample opportunities for germplasm resource collection (Laucou et al., 2011). While researchers have tirelessly worked to study the relative ability of different grape varieties to tolerate saline conditions, these efforts have not been comprehensive or systematic. As no unified standards for identifying salt-tolerant cultivars have been established and cultivation conditions may vary across studies, comparing the results of these different analyses is often not possible. There is thus a clear need to establish a precise high-throughput platform for analyzing grapevine germplasm salt tolerance, as such a system would enable to more effective identification of the raw materials needed to breed cultivars and rootstock varieties better equipped to tolerate soil salinization period.

## Identification of salt tolerance-related grape genes

Exposure to salt stress subjected plants to simultaneous ionic and osmotic stress, with relevant grapevine research conducted to date having largely focused on osmotic responses, ion accumulation, and the physiological characteristics of tolerant tissues. Under high levels of salt stress, osmotic changes occur rapidly with a half-time to the inhibition of Arabidopsis seedling root conductivity of just 45 min in response to 100 mM NaCl (Boursiac et al., 2005). Ion accumulation and osmoprotectant production can alter the ability of plant cells to balance water retention, and the aquaporin family of water channel proteins have been identified as particularly important regulators of plant salt stress responses (Li et al., 2014a). Further, plants would induce salt stress signaling pathway to decrease the adverse effects of excess Na^+^ and other ions. For instance, Ca^2+^, phytohormone (abscisic acid, ethylene, salicylic acid etc.), reactive oxygen species and related cascade signal transduction reactions will be activated to adapt the salinity environment (Zhao et al., 2021).

Grapevines exhibiting high levels of soluble sugar and proline accumulation reportedly exhibit less severe growth inhibition and higher leaf chlorophyll and carotenoid concentrations (Fozouni et al., 2012). A range of other genes have also been shown to be induced in cultivars exposed to salt stress including glycine betaine-associated genes and genes encoding proteins that are abundant during the late phases of embryogenesis including *VvDNHN1* and *VvLEA-D29L* (İbrahime et al., 2019; Haider et al., 2019). Multiple aquaporins have also been reported to play a role in osmotic regulation and salt tolerance in grapevines at the cellular and whole-plant levels (Galmés et al., 2007; Vandeleur et al., 2009). However, the fine regulation mechanisms of the aquaporins are still unclear and the expression pattern of some genes is ecotype-dependent. For instance, *VvPIP2;2* expression has been shown to be induced in salt-sensitive Shirazi plants, yet its expression is inhibited in salt-tolerant Gharashani plants (Mohammadkhani et al., 2012).

While some grape varieties are better able than others to exclude salt ions, the ion concentrations in these plants will inevitably be higher when cultivated in saline soil as compared to non-saline soil. Accordingly, these plants must engage a series of processes at the molecular level to adapt to or mitigate this ionic stress. Protein kinases and transcription factors have been shown to be particularly important proteins that can integrate inputs from multiple ion homeostasis- and stress signaling-related pathways in *Vitis*. *VaCPK21* was reportedly significantly up-regulated in wild grape (*Vitis amurensis* Rupr.) plants exposed to salt stress, with improved salt tolerance following the overexpression of this gene in grape callus samples (Dubrovina et al., 2016). Ji et al. found that *VvMAPK9* can serve as a positive regulator of Arabidopsis and grape callus adaptability through its ability to regulate antioxidant system activity. The highest levels of *VvMAPK9* expression were observed in root and leaf tissues, with pronounced induction in grapes in response to abscisic acid (ABA) or abiotic stressors such as high temperatures, salt, or drought conditions. Arabidopsis seedlings overexpressing *VvMAPK9* exhibited enhanced salt tolerance, and the germination rates of transgenic lines were higher, with these plants exhibiting superior growth and longer roots under salt stress conditions as compared to wild-type plants. The expression of antioxidant enzyme (SOD and POD)s and ion transporter-related proteins (NHXP, HKT1, HKT2) was also significantly elevated in these *VvMAPK9-*overexpressing grape callus samples under salt stress conditions (Ji et al., 2022).

Transcription factors are expressed in all eukaryotic species and are essential regulators of signaling activity in plants exposed to abiotic stress conditions, promoting the upregulation of stress resistance-related genes such that they are an important focus of research exploring stress tolerance in plants. A transcriptomic analysis of grapes exposed to salt stress identified 52 transcription factors including WRKYs, EREBs, MYBs, NACs, and bHLHs among the 343 differentially expressed genes (Upadhyay et al., 2018). The C-repeat (CRT)/dehydration-responsive element (DRE) protein family is comprised of key regulators of plant abiotic stress tolerance, and the CRT/DRE binding factor *VaCBF4* can be induced in *V. amurensis* in response to cold, drought, ABA, saline conditions, and other abiotic stressors, improving the ability of Arabidopsis seedlings to tolerate cold, drought, and salt stress when overexpressed (Li et al., 2013). VvWRKY30 is a transcription factor that is primarily expressed in leaves and shoot tips and that can be induced in response to salt stress, H_2_S, and H_2_O_2_, enhancing the ability of plant seedlings to tolerant saline conditions through the enhanced elimination of reactive oxygen species and osmotic membrane accumulation. When *VvWRKY30* is overexpressed, seedlings reportedly exhibit improved antioxidant activity and corresponding reductions in reactive oxygen species, together with increases in soluble sugar and proline content and the concomitant upregulation of genes associated with antioxidant biosynthesis, sugar metabolism, and proline biosynthesis under salt stress conditions (Zhu et al., 2019). The *VviERF073* transcription factor is a member of the APETALA2/ethylene response factor (*AP2/ERF*) family firstly identified as a salt stress-inducible gene in a salt stress EST library, although subsequent reports of its functional role in grape plants exposed to salt stress conditions have been lacking (Shinde et al., 2017). The helix-loop-helix transcription factor *VvbHLH1* can significantly enhance flavonoid accumulation within Arabidopsis seedlings when overexpressed in a codon-optimized isoform, with further research suggesting that it can also shape drought and salt tolerance in Arabidopsis plants through the augmentation of ABA signal transduction and flavonoid accumulation (Wang et al., 2016). *VpSBP16* encodes a SQUAMOSA promoter binding protein (SBP) box transcription factor that was cloned from the Chinese wild grape ‘Baihe 35-1’ variety that was found to regulate SOS and ROS signaling cascades to improve salt and drought stress tolerance during seed germination. Consistently, transgenic Arabidopsis seedlings in which *VpSBP16* was overexpressed exhibited increased root length and seed germination rates as compared to wild-type plants exposed to osmotic stress (Hou et al., 2018).

Under salt stress conditions, Na^+^/H^+^ antiporter (NHX) proteins can facilitate the ATP-dependent transport and sequestration of Na^+^, thereby effectively mitigating ionic stress in plants. The *AtNHX1* gene reportedly conferred ‘Thompson seedless’ grape plants with a level of salt tolerance similar to that observed for other salt-tolerant cultivars (‘Pedro Gimenez’ and ‘Criolla Chica’). ‘Thompson seedless’ seedlings overexpressing the Arabidopsis-derived *AtNHX1* gene exhibited growth that was better than that of wild-type seedlings when treated for 7 weeks with 150 mM NaCl including significant improvements in stem length, leaf area, and dry weight (Venier et al., 2018). When overexpressed in potato seedlings, grape-derived *VvNHX1* was similarly able to improve salt tolerance. These transgenic seedlings also reportedly exhibited higher levels of soluble sugar, Mg^2+^, and K^+^, together with enhanced antioxidant enzyme activity and reductions in Na^+^ accumulation and oxidative stress (Li et al., 2014b; Charfeddine et al., 2019).

## Cultivation practices that can improve the salt tolerance of grape plants

The application of optimized cultivation and management practices has the potential to improve the ability of grape plants to tolerate salt stress. Appropriately managing soil and water can help decrease the adverse effects of saline conditions on grape plants. Low ABA concentrations, for example, can render cells better able to adapt to saline conditions while reducing transpiration and the passive absorption of salt ions, supporting a link between ABA accumulation and the expression of functional genes including *VvNHX1* and *VvOSM1* (Saleh et al., 2020). Vineyard management technologies can help alleviate salinization by prolonging irrigation time to leach Na^+^ and Cl^-^ accumulated in the root zone, or by artificial ditches to induce rainfall inflow to leach salt in the soil below a depth of 1.5 m in the ground. The partial root drying (PRD) technique was designed to optimize the efficiency of water use for viticulture by reducing irrigation while improving fruit quality (Dry et al., 2000). Degaris et al. explored the effects of moderately saline water on Shiraz and Grenache vines grown in pots using the PRD irrigation technique, which reduces the amount of water used by 50% relative to control conditions (Degaris et al., 2016). Their results suggested that PRD-irrigated vines exhibited higher levels of Cl^-^, Na^+^, K^+^, and Ca^2+^ ions, but that Cl^-^ can be partitioned away from leaves on a total content basis relative to controls. These results demonstrate that combining PRD irrigation techniques and saline water can alter ion levels and allocation within grapevines, underscoring the need to monitor field water during the growing season to promote long-term vine health and improved fruit composition.

ABA can reportedly induce phytochemical and morphological changes that can enhance the ability of grape plants to tolerate salt stress. Grape rootstocks with superior tolerance characteristics have been found to accumulate higher levels of ABA when exposed to salt stress (Upreti and Murti, 2010). Relative to untreated seedlings, seedlings subjected to exogenous ABA treatment exhibited increases in plant height, leaf area, leaf number, and shoot dry matter together with increases in leaf flavonoid, proline, soluble sugar, and phenol levels and enhanced activity of antioxidant enzymes including catalase, guaiacol peroxidase, and ascorbic acid peroxidase (Stevens et al., 2011). Acetic acid and oxalic acid irrigation can also promote significant increases in the root activity and leaf chlorophyll content of treated ‘Fuke’ cuttings exposed to salt stress while reducing root malondialdehyde levels and leaf H_2_O_2_ concentrations (Guo et al., 2018).

## Discussion

Soil salinization represents an increasingly severe threat to agronomic productivity throughout the globe, endangering the reliable production of foods necessary for human survival. Most popular grape varieties cultivated at present are of European provenance, but many of these exhibit relatively low poor tolerance for soil salinity. Further efforts to leverage stress-resistant wild grape germplasm resources thus have the potential to provide key genes and rootstocks needed to breed salt-tolerant grape varieties. *V. riparia*, *V. champini*, *V. berlandier*, and *V. shuttleworthii* are salt-resistant North American grape varieties that represent promising rootstock materials for the further breeding of salt-tolerant varieties. Further efforts to understand the physiological and ecological characteristics and mechanisms governing rootstock salt tolerance will offer a scientific basis for the more effective breeding and cultivation of grape varieties that can tolerate rising levels of soil salinity.

Recent studies of grape salt tolerance have largely centered around efforts to evaluate germplasm resources, characterize physiological salt tolerance mechanisms, and related topics, providing a foundation for the breeding of salt-tolerant grapes. However, insufficient work focused on the isolation, cloning, and regulated expression of salt tolerance-related grape genes has been performed to date. There is thus an urgent need to develop biotechnology-based approaches to enhancing the adaptability of grape plants to salinized soil. The ongoing development of proteomics and functional genomics platforms, together with the application of novel technologies such as expressed sequence tags, cDNA microarrays, transposon tags, and T-DNA tags provide new opportunities for the more straightforward isolation and characterization of salt tolerance-associated genes in the future.

Based on the findings reported in this review, we believe that research evaluating salt-tolerant grape germplasm resources is still in its early stages with a clear lack of systematic research efforts. It is therefore crucial that a salt-tolerant germplasm resource database be established to provide robust data that can support breeding efforts. Wild grape resources are widely distributed and represent a rich source of novel genetic elements that warrant a higher degree of attention. These ongoing efforts to identify key salt tolerance-related genes and to more fully outline the functional relationship between salt stress and signaling activity in wild grapes may provide a foundation for the targeted breeding of salt-tolerant grapes through the appropriate application of genomics and proteomics techniques.

To date, studies focused on identifying salt-tolerant grape germplasm resources have largely been restricted to laboratory settings, since NaCl irrigation is generally used to simulate soil salinization as a means of testing seedling or seed responses to a range of salt concentrations. These artificial conditions, however, differ from true soil salinity. Tests of salt tolerance-related gene functions have also primarily been conducted in Arabidopsis model plants, limiting efforts to comprehensively survey and validate salt tolerance in grape germplasm resources. As such, while these prior findings provide a valuable foundation for further research efforts, they must be interpreted with caution owing to these limitations, underscoring the need for additional systematic physiological research efforts to better expand the current understanding of salt tolerance in grape cultivars.

## Author contributions

YH: Conceptualization, Project administration, Writing – original draft, Writing – review & editing. XL: Conceptualization, Funding acquisition, Investigation, Supervision, Writing – original draft, Writing – review & editing.
